# Basal ganglia and cerebellar lesions causally impact the neural encoding of temporal regularities

**DOI:** 10.1162/imag_a_00492

**Published:** 2025-02-27

**Authors:** Antonio Criscuolo, Michael Schwartze, Sylvie Nozaradan, Sonja A. Kotz

**Affiliations:** Department of Neuropsychology & Psychopharmacology, Faculty of Psychology and Neuroscience, Maastricht University, Maastricht, the Netherlands; MARCS Institute for Brain, Behaviour and Development, Western Sydney University, Sydney, Australia; International Laboratory for Brain, Music and Sound Research (BRAMS), Montreal, Canada; School of Health and Behavioural Sciences, University of the Sunshine Coast, Queensland, Australia; Department of Neuropsychology, Max Planck Institute for Human Cognitive and Brain Sciences, Leipzig, Germany

**Keywords:** timing, rhythm, oscillations, neural dynamics, basal ganglia, cerebellum

## Abstract

Acting in and adapting to a dynamically changing environment necessitates to precisely encode the*timing*of sensory events, and to*time*our own (re-)actions to them. Cerebellar (CE) and basal ganglia (BG) circuitries play fundamental and complementary roles in timing processes. While the CE seems to use precise timing (*when*an event occurs) and temporal intervals to generate temporal predictions (*when*a next event occurs), the BG uses relative timing to extract the beat in rhythmic sequences. As it is generally difficult to record data from respective patient groups in parallel, CE and BG contributions to timing processes are rarely investigated in combination. Here, we let healthy controls and patients with CE or BG lesions listen to isochronous auditory sequences while their EEG was recorded. We assessed intra- and inter-individual variabilities, as well as group differences, using event-related potentials (ERP), delta-band inter-trial phase-coherence, and acceleration dynamics while tuning to the stimulation frequency (*Sf*). CE and BG lesions increased variability in ERP latency and reduced the coherence of delta-band activity. CE but not BG lesions further impacted the stability of delta-band oscillations while tuning to the*Sf*. These findings show a causal link between subcortical lesions and the capacity to encode and synchronize ongoing neural activity with temporal regularities in the acoustic environment. While most*standard*metrics of neural entrainment do not dissociate specific contributions of BG and CE to sound processing in isochronous sequences, the newly introduced ‘stability’ metric isolated distinct changes in delta-band tuning dynamics in CE patients. This observation highlights the fundamental role of the CE in generating and maintaining stable neural representations of event onsets in the sensory environment.

## 1. Introduction

Time is a fundamental dimension of human cognition. Every decision, every action, every sensory stimulus around us happens in time and at multiple timescales. Our capacities to encode the precise*timing*of events and to*time*our (re-)actions to them are pivotal for acting in and adapting to a continuously changing dynamic environment. The environment displays some gradient of*temporal regularity*(Greenfield, Honing, et al., 2021): there are various periodicities within the body (e.g., the heartbeat and respiration;Criscuolo, Schwartze, et al., 2022) as well as in simple and complex sensory input (e.g., music, speech) and in behavior (e.g., interpersonal interactions in the animal and human world;Greenfield, Aihara, et al., 2021). These temporal regularities (or*rhythm*) can facilitate the processing of the*timing*of sensory events and further enable individuals to anticipate the*when*of next sensory events. In turn, temporal anticipation can benefit*adaptive*behavior: we can dance to music because we know*when*the next beat falls, and we can synchronize with others because their movement timing is predictable. Yet what determines our capacity to adapt behavior in time? Do we differ in the*if*and*how*we adapt and synchronize with auditory rhythms?

Human (e.g.,Schroeder & Lakatos, 2009) as well as nonhuman animals (e.g.,Criscuolo, Schwartze, Prado et al., 2023;Lakatos et al., 2008) encode and synchronize with temporal regularities by generating temporal predictions (Criscuolo, Schwartze, Prado et al., 2023;Lakatos et al., 2005). Temporal predictions allow to estimate the*when*next events occur, thus enabling*dynamic attending*(Large & Jones, 1999) of event onsets. Temporal predictions materialize as anticipatory neural activity (Arnal, 2012;Breska & Deouell, 2017;Fujioka et al., 2009,^2012^,^2015^;Ross et al., 2018;Snyder & Large, 2005), which supports the predictive alignment of neural oscillations to event onsets, fosters sensory processing (Lakatos et al., 2013), and further allows for production and synchronization of behavior in a predictive manner (Bartolo & Merchant, 2015;Gámez et al., 2018). Thus, ‘adaptation by anticipation’ (Fraisse, 1963; p. 18) is a mechanism for optimizing behavior and operates across sensory modalities (Arnal & Giraud, 2012;Cravo et al., 2013;Friston, 2005;Morillon et al., 2016;Nobre et al., 2012).

Fundamental to predictive temporal processes is the engagement of an extended motor system (Arnal, 2012;Fujioka et al., 2012,^2015^;Schwartze & Kotz, 2013), recruited to prepare and execute predictive and synchronized behavior. The cerebellum (CE) and the basal ganglia (BG) are key structures in an extended cortico-subcortico-cortical brain network that plays a pivotal role in temporal processing (Merchant, Harrington, et al., 2013;Schwartze & Kotz, 2013). The CE encodes the precise timing (*when*an event onsets) of sensory events in the subsecond range (Bareš et al., 2018;Ivry et al., 1988;Ivry & Keele, 1989;Ivry & Schlerf, 2008) and thus allows estimating the duration of temporal intervals (Breska & Ivry, 2020,^2021^;Grube, Cooper, et al., 2010;Grube, Lee, et al., 2010;Teki, Grube, & Griffiths, 2011;Teki, Grube, Kumar, et al., 2011) by quantifying the time elapsed between event onsets. The BG support the generation of temporal predictions (*when*the*next*event occurs) and use relative timing to process the beat (Grahn, 2009;Grahn & Brett, 2009;Schwartze et al., 2011;Teki, Grube, Kumar, et al., 2011).

CE lesions alter the encoding of event onsets in complex sound sequences (Nozaradan et al., 2017), as well as responses to single acoustic events, as evidenced by delayed and variable early event-related responses in the EEG (Schwartze & Kotz, 2021). CE lesions also affect the capacity to predict temporal intervals (Breska & Ivry, 2018,^2020^,^2021^;Grube, Cooper, et al., 2010) and impact the ability to produce and synchronize with rhythms (Ivry & Keele, 1989;Ivry et al., 1988). However, temporal predictions are comparable to healthy controls when tested in rhythmic contexts (Breska & Ivry, 2018,^2020^,^2021^).

A reversed pattern was observed in BG lesion patients: while their interval discrimination seems intact, their performance deteriorated in rhythmic contexts (Breska & Ivry, 2018). This observation suggests a fine, yet fundamental dissociation between rhythm- and interval-based temporal predictions in BG and CE lesion patients (Breska & Ivry, 2018). In fact, patients with BG lesions are less sensitive to temporal regularity in auditory sequences, have difficulties in generating temporal predictions in both basic (Schwartze et al., 2015) and complex (syncopated rhythms;Nozaradan et al., 2017; music (Grahn & Brett, 2009) and speech (Kotz & Schmidt-Kassow, 2015)) auditory sequences, and in coordinating and adjusting their tapping to tempo changes (Schwartze et al., 2011). Altogether, observations suggest intact single interval-based timing in the BG patients (Breska & Ivry, 2018;Grube, Cooper, et al., 2010;Teki, Grube, Kumar, et al., 2011), but impaired processing of temporal regularity in complex sequences.

What underlies these dysfunctions? Can the analysis of neural oscillatory activity causally show in what way the cortico-subcortical timing network differentiates the processing of temporal regularities?

We addressed these questions in an EEG experiment involving healthy ageing controls (HC) and patients with focal lesions in either the CE or BG. Participants listened to isochronous auditory sequences while EEG was recorded. We expected CE patients to show increased variability in the temporal encoding of tone onsets, while we hypothesized BG patients to have difficulties in generating temporal predictions. Single-trial event-related potential (ERP) and inter-trial phase coherence (ITPC) analyses allowed testing if the participants’ neural activity responded in time to sound onsets, thus encoding their precise timing. Next, we employed a time-resolved metric of ITPC (t-ITPC) to quantify the build-up of phase-coherence over the course of the auditory sequence, an indicator of temporal prediction (Breska & Ivry, 2020) and auditory processing (Ten Oever et al., 2017). Finally, we quantified the*tuning*of delta-band neural oscillatory activity toward the stimulation frequency (*Sf*). Thus, we estimated trial-level instantaneous frequency (IF), its dynamics of acceleration while tuning- and de-tuning to the Sf, its Stability (S), and Deviation (Dev) from the Sf. These newly introduced metrics would allow to estimate*if*and*how much*endogenous neural activity in the delta-band fluctuates around the prominent frequency in the acoustic stimulation. As such, it would provide new insights on the time-varying neural dynamics of entrainment while listening to temporally regular auditory rhythms.

## 2 Materials & Methods

### 2.1 Participants

Thirty-three participants took part in the study and signed written informed consent in accordance with the guidelines of the ethics committee of the University of Leipzig and the declaration of Helsinki. Participants comprised two patient groups and one age- and gender-matched control group: 11 patients with focal lesions in the basal ganglia (BG; mean age 50.9, range = 30–64 years; 5 males), 11 patients with focal lesions in the cerebellum (CE; mean age 52.6, range = 37–64 years; 5 males), and 11 healthy controls (HC; mean age 52.1, range = 28–63 years; 5 males). Patients’ demographics and lesion information are provided inTable 1, and their anatomical MRI is provided inFigure 1.

**Table 1.** Individual patient history.

CE	Age	Location	Additional locations	Etiology	Volume in cc	X	Y	Z
	64	Posterior cerebellar lobule	None	PICA infarction	7,1	89	37	26
	30	Posterior cerebellar lobule	Superior frontal gyrus, corpus callosum	ICH and AVM	48	77	38	35
	49	Posterior cerebellar lobule, tonsil, vermis	None	PICA infarction	14,5	57	40	24
	53	Anterior and medial cerebellar lobule, tonsil	None	PICA infarction	7,9	62	41	24
	39	Anterior and posterior cerebellar lobule	None	cyst extripation	2,4	88	52	15
	62	Posterior cerebellar lobule	Thalamus	PICA infarction	9	111	69	37
	62	Posterior cerebellar lobule	Telencephalic white matter	PICA infarction	3,1	57	58	55
	59	Posterior cerebellar lobule, tonsil	None	PICA infarction	3,6	110	38	30
	63	Posterior cerebellar lobule	Telencephalic white matter	PICA infarction	5,7	104	61	46
	37	Vermis, deep cerebellar nuclei, peduncle	None	tumor postoperatively	8,8	84	49	37
	42	Posterior cerebellar lobule	None	PICA infarction	0,3	121	48	27
*BG*								
	41	Putamen, pallidum, caudate body, IC	Corona radiata	MCA infarction	23,4	56	113	76
	59	Putamen, pallidum, caudate head and body, IC	Corona radiata	ICH	18,3	53	119	76
	52	Putamen	None	ICH	2,8	104	99	74
	61	Claustrum, putamen, pallidum, caudate body, EC, IC	Corona radiata, thalamus	ICH	8,8	51	98	77
	37	Putamen, caudate body, IC	None	MCA infarction	0,8	55	97	88
	50	Putamen, caudate body, IC	Corona radiata, corpus callosum	MCA infarction	6,7	54	110	81
	60	Putamen	None	MCA infarction	0,5	47	102	64
	55	Putamen, pallidum, caudate body	None	MCA infraction	3,0	102	101	77
	51	Claustrum, putamen, EC	Insula	ICH	14,8	106	102	78
	64	Putamen, pallidum, caudate body, IC	None	MCA infarction	6,1	98	116	80
	49	Putamen, caudate body	Thalamus	MCA infarction	6,3	93	103	82

In order, from left to right, age, lesion location, secondary lesion location, lesion etiology, volume of the lesion in cc, and lesion coordinates for the center of mass (as provided by MRIcron). Top for cerebellar (CE) patients, and bottom for basal ganglia (BG) patients.

Abbreviations: PICA = posterior cerebellar artery, ICH = intracerebral/-cerebellar hemorrhage, AVM = arteriovenous malformation, IC = internal capsule, EC = external capsule, MCA = Middle cerebral artery.

**Fig. 1.**
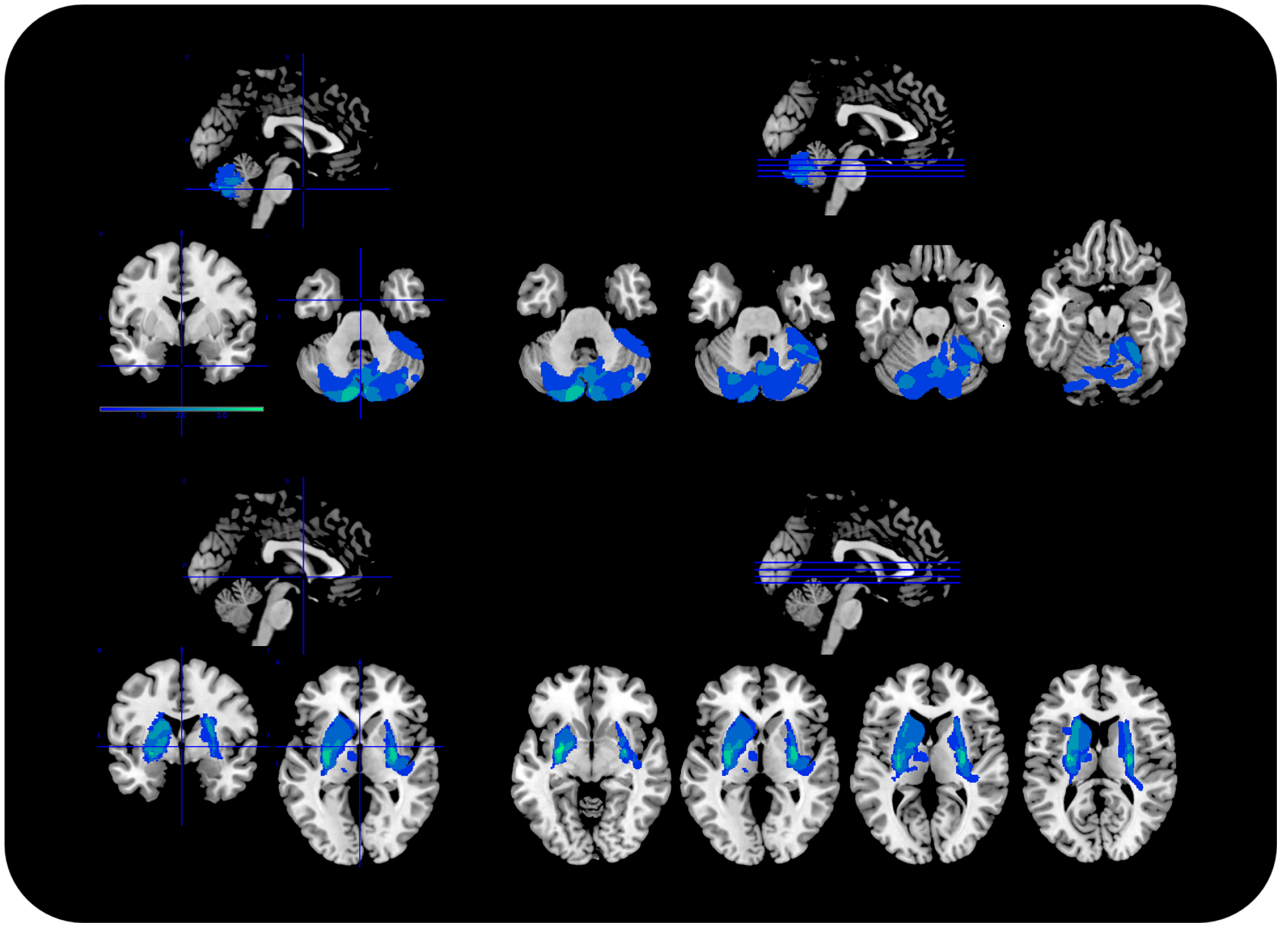
Brain lesion delineation and overlap. The figure provides the brain lesion delineation on a template anatomical MRI for cerebellar (CE; top) and basal ganglia (BG; bottom) patients. The figures were obtained by using MRIcron (http://www.mccauslandcenter.sc.edu/mricro/mricron/), and display the lesion overlap color-coded in shades of blue (blue = minimal overlap; light blue high overlap; values range from 0 to 5).

The HC group was recruited via a database at the Max Planck Institute for Human Cognitive and Brain Sciences (Leipzig, Germany). All participants were right-handed and matched for years of education. None of the participants were professional musicians, and they reported no history of hearing or psychiatric disorders. All participants received monetary compensation for taking part in the study. Further information on the participants and lesions characteristics can be found inNozaradan et al. (2017).

### 2.2 EEG experiment: design and procedure

**Fig. 2.**
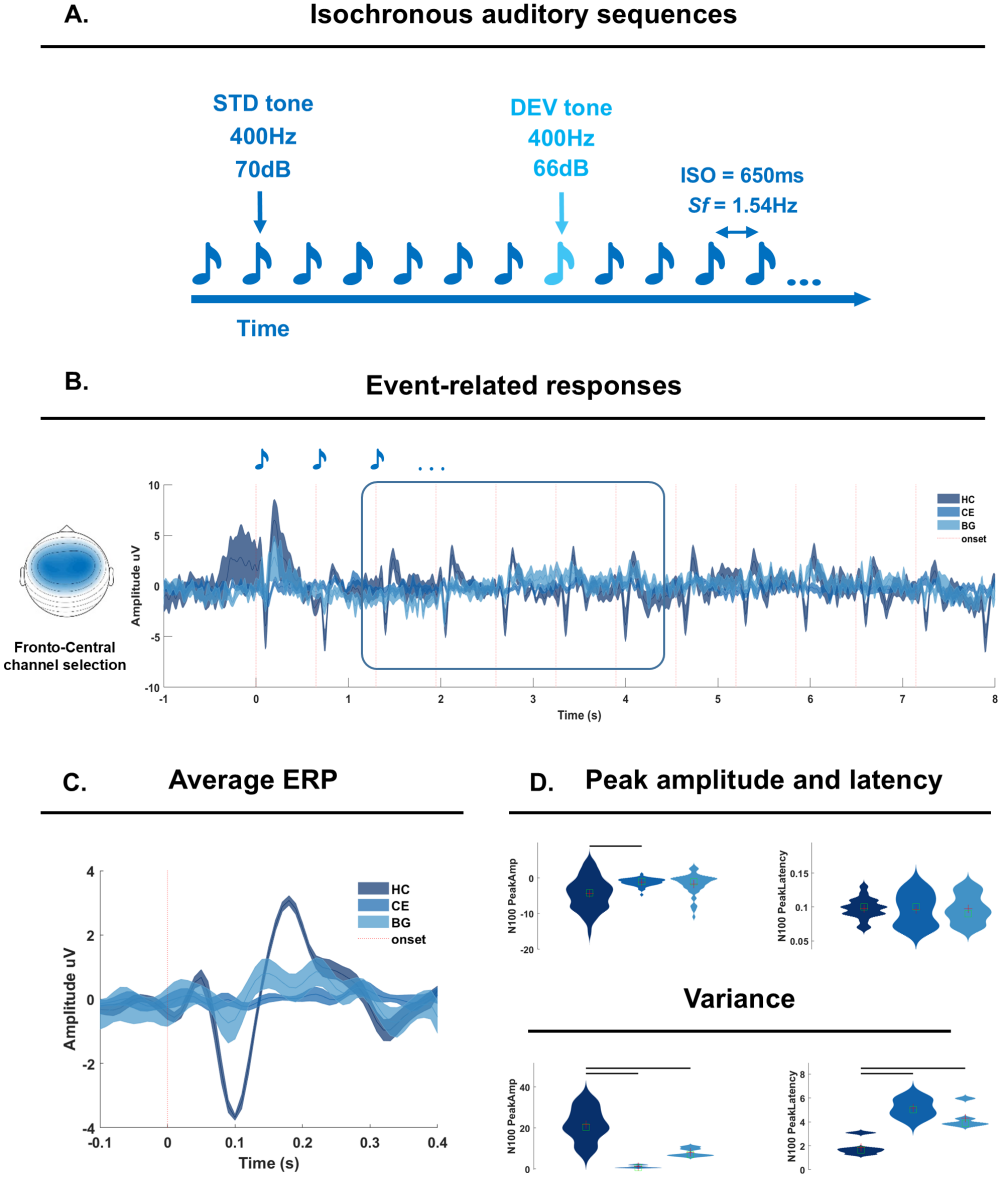
EEG experiment and ERP analyses. (A) Experimental design: participants listened to 96 isochronous auditory sequences presented at a fixed Stimulation frequency (Sf) of 1.54 Hz (inter-stimulus onset (ISO) of 650 ms). The sequences included frequent standard (STD; pitch 400 Hz, loudness 70 dB) and infrequent amplitude deviant (DEV) tone (amplitude 66 dB). (B) Sequence-level ERPs as calculated in a fronto-central channel cluster (left) and color coded per group (dark blue for HC; light blue for BG; medium blue for CE). Shades report the standard error calculated across participant. (C) Tone-level ERP, obtained by averaging across sound repetitions, and among tones in the 3rd to 7th position (as highlighted by the rectangle in B). Shades report the standard error calculated across participant. (D) Group-level peak amplitude (top left) and latency (top right). Below, group-level variance in peak amplitude and latency. Red crosses and green squares report mean and median respectively. Horizontal lines report a significant group effect and significant pairwise comparisons (seeSuppl. Tables 2, 3, 4).

Participants listened to 96 sequences comprising 13-to-16 tones in a recording session of approximately 25 min. Each sequence contained frequent standard tones (STD; F0 = 400 Hz, duration = 50 ms, rise and fall times = 10 ms, amplitude = 70 dB SPL) and one or two amplitude-attenuated deviant tones (DEV; amplitude 66 dB). The inter-onset-interval between successive tones was 650 ms, resulting in a stimulation frequency (*Sf*) of 1.54 Hz, and a total sequence duration of 8.45–10.4 s (13 to 16 tones * 650 ms;Fig. 2A).

Participants were seated in a dimly lit soundproof chamber facing a computer screen. Every trial started with a fixation cross (500 ms), followed by the presentation of an auditory sequence. The cross displayed on the screen served to prevent excessive eye movements during the presentation of the tone sequences. After each sequence, there was an inter-trial interval of 2000 ms. A session was divided into two blocks of approximately 10 min each, with a short pause in between. At the end of the experiment, participants indicated how many softer tones they heard.

#### 2.2.1 EEG recording

The EEG was recorded by means of 59 Ag/AgCl scalp electrodes positioned according to the International 10-10 system with the ground placed on the sternum. Four additional vertical and horizontal electrodes monitored eye movements and were positioned on the outer canthus of each eye, and on the inferior and superior areas of the left orbit. The signals were amplified, low-pass filtered at 512 Hz, and digitized using a sampling rate of 1024 Hz (64-channel high-speed amplifier, Biosemi, the Netherlands). Electrode impedances were kept below 5 kΩ, and the left mastoid served as online reference. Data were referenced to an average reference offline.

#### 2.2.2 Data analysis

##### 2.2.2.1 EEG preprocessing

EEG data were analyzed in MATLAB with a combination of custom scripts and functions and the FieldTrip toolbox (Oostenveld et al., 2011). Data were band-pass filtered with a 4th order Butterworth filter in the frequency range of .1–50 Hz (*ft_preprocessing*). Eye-blinks and other artifacts were identified using independent component analysis. This semi-automated routine is composed of two steps: in the first iteration, ‘*fastICA’*(implemented in FieldTrip) was applied to decompose the original EEG signal into independent components (N = number of EEG channels -1). Components with a strong correlation (>.4) with the EOG time-courses were automatically identified and removed with ‘*ft_rejectcomponent*’ before reconstructing the EEG time-course. In a second step, ‘*fastICA*’ was used again*,*now with a dimensionality reduction to 20 components. These components were visually inspected via ‘ft_*rejectvisual*’ and marked as ‘outliers’ if their max values and z-scores were far from the distribution of other components. The 20 components were further visually inspected by plotting their topographies and time-series, and a second selection of ‘outliers’ was made. Taking into consideration the two visual inspections, we made a final decision on which components to remove. On average, two components were removed (‘*ft_rejectcomponent*’) before reconstructing the EEG time-series. In the next preprocessing step, artifact subspace reconstruction was performed as implemented in the ‘*pop_clean_rawdata*’ function in EEGlab, and with the ‘BurstCriterion’ parameter set to 20 (as recommended in the online EEGlab tutorials; all other parameters were set to ‘off’). To further ensure the removal of potentially noisy channels and time-points, we implemented an automatic channel rejection and an artifact suppression procedure. To this end, the median variance across channels was calculated (excluding EOG channels), and channels exceeding 2.5*median variance were defined as ‘outliers’ and removed. Similarly, the artifact suppression procedure (seeCriscuolo, Schwartze, Henry, et al., 2023) interpolated noisy (>4*absolute median) time-windows on a channel-by-channel basis. Lastly, data were low-pass filtered at 40 Hz via ‘*ft_preprocessing*’, segmented to each auditory sequence (starting 4 s before the first tone onset and ending 4 s after the last tone onset), and downsampled to 250 Hz. All the following analyses focused on a region of interest. As in prior work (Criscuolo, Schwartze, Henry, et al., 2023), a fronto-central channel (FC) cluster was used, encompassing the sensor-level correspondents of prefrontal, pre-, para-, and post-central regions highlighted in (Fujioka et al., 2012,^2015^) and further highlighted in similar EEG work on rhythm processing (Nozaradan et al., 2012). The cluster included 14 channels: ‘AFz’, ‘AF3’, ‘AF4’, ‘F3’, ‘F4’, ‘F5’, ‘F6’, ‘FCz’, ‘FC3’, ‘FC4’, ‘FC5’, ‘FC6’, ‘C3’, ‘C4’.

##### 2.2.2.2 ERP analyses

To perform ERP peak analyses, preprocessed data were low-pass filtered at 30 Hz via ‘*ft_preprocessing*’ and averaged across FC channels. ERP time-series are provided inFigure 2B, and are color-coded for each group. Next, these time-series were segmented to each tone onset in the auditory sequences (average over trials provided inFig. 2C) and mean corrected. The mean correction uses a global mean calculated across the whole segment (-.1 to .4 s relative to tone onset) and averages across all trials (Criscuolo, Schwartze, Henry, et al., 2023,Criscuolo, et al., 2024). To perform peak analyses, we set out to identify, at the single participant level, the N100 peak amplitude and its latency for each tone onset, after averaging across sequences. We created a window of interest in the time interval between .07 s and .13 s relative to tone onsets and identified the most negative amplitude value (N100 peak amplitude) and its latency (N100 peak latency). Finally, we calculated, at the single-participant level, the variance in N100 amplitude and latency across tone positions. Peak amplitude, latency, and variance are provided inFigure 2D, pooling single-participant data per group. Analyses focused on the 3rd to the 7th tone: the first two sound onsets were excluded as they are known to trigger much stronger ERPs; the later tones are excluded because they occur in proximity of the DEV (8th position onward). Analyses on the first two tones in the sequence, where no group differences were expected, are provided inSuppl. Figure 1.

###### 2.2.2.2.1 Mixed effect models on ERP data

We assessed group differences in the N100 peak amplitude and latency via Mixed effect models (‘*fitlme*’ function in MATLAB, version 2024). The model included ‘Group’ as a fixed factor and a random intercept per participant. When the fixed effect was significant, we performed post-hoc pairwise group comparisons via permutation testing. Pair-wise comparisons were thus performed by randomly permuting data points belonging to one or the other group, with a total of 1000 permutations. This iterative procedure would ultimately assess the p-value from the original groups against the p obtained from permutations. Model information and results are reported inSuppl. Tables 1-2.

###### 2.2.2.2.2 Statistical comparisons on the variance

We assessed the variability in the N100 peak amplitude and latency across groups. For these two analyses, we first performed Levene’s test to assess the homogeneity of variance and later employed ANOVA. With a significant group effect, we later performed post-hoc pairwise comparisons via simple t-tests and implementing the Tukey-Kramer correction. Analyses details and results are provided inSuppl. Tables 3-4.

##### 2.2.2.3 Inter-trial phase coherence

**Fig. 3.**
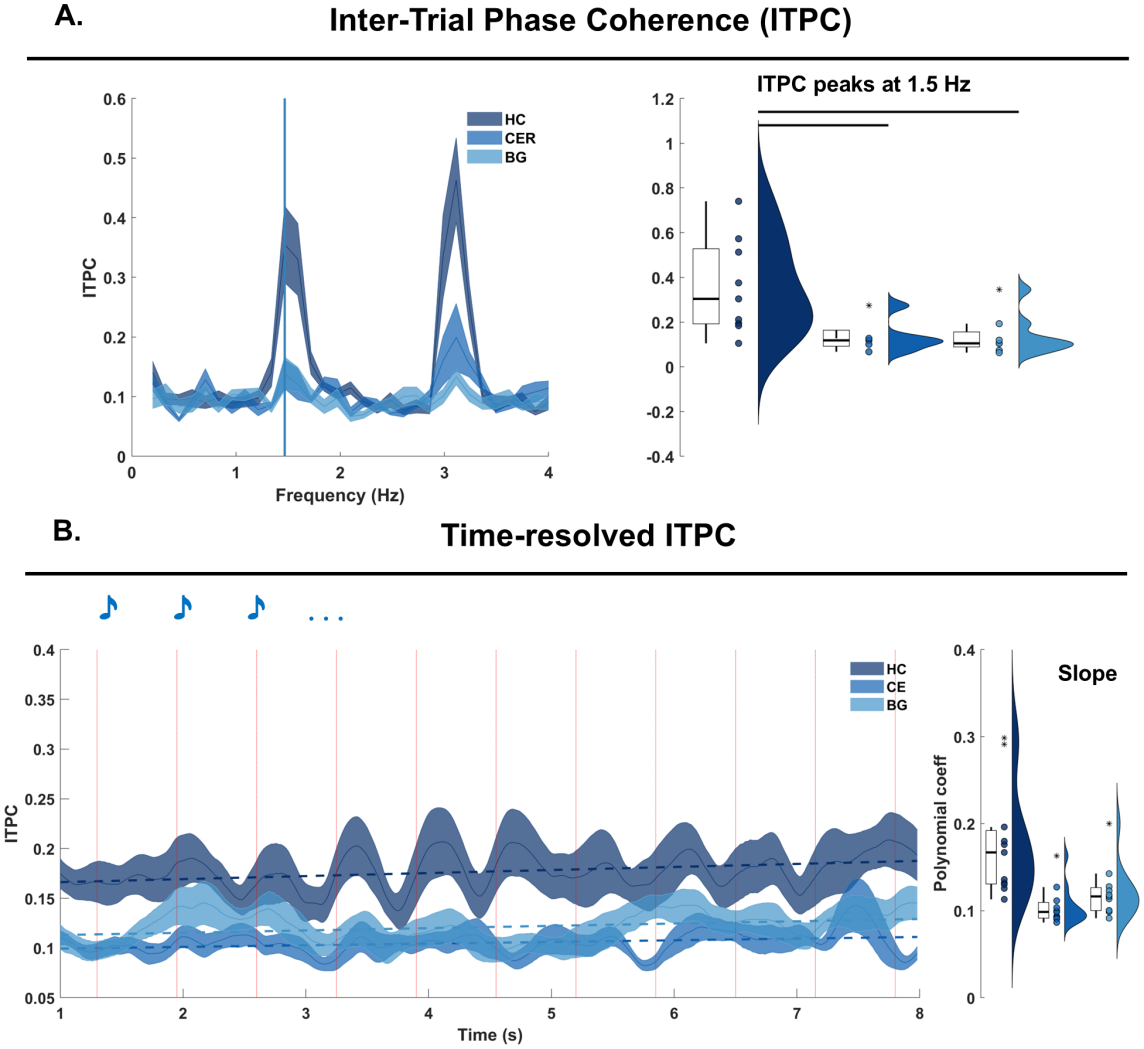
Inter-Trial Phase Coherence (ITPC) analyses. (A) Inter-Trial Phase Coherence (ITPC) analyses. The ITPC plot features frequency in Hz on the x-axis and the ITPC values on the y-axis. Healthy controls (HC), cerebellar (CE) and basal ganglia (BG) patients’ data are provided in shades of blue (from dark to light blue; solid line reports the mean across participants, and shades represent the standard error across participants). The vertical blue line signals the ITPC peak at 1.5 Hz. These peaks were extracted and plotted on the right; each dot corresponds to a single participant. Here, statistical comparisons assessed group differences in ITPC values at the stimulation frequency (1.5 Hz). The black horizontal lines indicate significant differences between the groups, after correction for multiple comparisons. (B) Time-resolved ITPC was computed from the complex spectra of continuous wavelet transformed data. Shades report the standard error calculated across participants. The left plot features the time-course of t-ITPC in the time-range between 1-8 s (x-axis) and the ITPC value on the y-axis. On top, the dashed lines represent the slope of each time-course. Each participant’s slope is then plotted in the distribution on the right side, showing polynomial coefficient (y-axis) per group (color coded).

Inter-trial phase coherence (ITPC) analyses were conducted to test whether healthy participants and patients encoded temporal regularities in the auditory sequences. To estimate ITPC, we first performed Fast-Fourier transform (FFT). FFT analyses were performed at the single-participant, -channel, and -trial level on 8-s-long segments starting from the onset of the first tone in the auditory sequence and including a total of 12 tones. The resulting frequency resolution was .125 Hz (1/8 s = .125 Hz). As in prior work (Criscuolo, Schwartze, Henry, et al., 2023), and for the ERP analyses described above, a fronto-central channel (FC) cluster was used, including the following 14 channels: ‘AFz’, ‘AF3’, ‘AF4’, ‘F3’, ‘F4’, ‘F5’, ‘F6’, ‘FCz’, ‘FC3’, ‘FC4’, ‘FC5’, ‘FC6’, ‘C3’, and ‘C4’. Data from this FC cluster were not averaged at this stage. Next, the complex part of the Fourier spectrum was used to calculate ITPC (Fig. 3Aleft). ITPC was obtained by dividing the Fourier coefficients by their absolute values (thus, normalizing the values to be on the unit circle), averaging, and finally taking the absolute value of the complex mean (for further documentation seehttps://www.fieldtriptoolbox.org/faq/itc/). For illustration purposes, the ITPC plot inFigure 3Ais restricted to 1–4 Hz. The full frequency spectrum is provided inSuppl. Figure 2, along with the Fourier spectrum in the frequency-range of 4–30 Hz.

As the stimulation frequency of the auditory sequences was 1.5 Hz, we restricted the main ITPC analyses to 1.5 Hz only. We did not conduct statistical analyses on any other frequencies (e.g., (sub)harmonics of the stimulation frequency) because we were specifically interested in assessing the neurophysiological encoding of the auditory rhythm at the stimulation frequency as previously reported in (Criscuolo, Schwartze, Henry, et al., 2023,Criscuolo, Schwartze, Prado, et al., 2023). Thus, single-participant data were pooled per group after averaging across channels and visualized as violin plots inFigure 3A(right).

###### 2.2.2.3.1 Statistical comparisons

Group differences were statistically assessed by means of a 1-way ANOVA with a group factor (*anova1*built-in in MATLAB), followed by post-hoc simple tests corrected for multiple comparisons via Tukey-Davis correction (*multcompare*built-in in MATLAB) in case of a significant (*p*< .05) main effect (Suppl. Table 5). Simple effects with a*p-*value below an alpha-corrected .05 were considered statistically significant. The choice of the parametric test was informed by Levene’s test for homogeneity of variance (Suppl. Table 5).

Additional analyses were performed to test the hypothesis that group differences are specific to the rhythm of the auditory sequences. We statistically assessed group differences in the alpha (8–12 Hz) frequency band following the same statistical procedure as detailed above, for both the ITPC and Fourier spectrum (Suppl. Fig. 2).

##### 2.2.2.4 Time-resolved ITPC

A time-resolved metric of ITPC (t-ITPC) was estimated to quantify the build-up of phase-coherence over the course of an auditory sequence. For doing so, we first employed time-frequency transform (TF data), then calculated the t-ITPC using the complex spectra of TF data (same procedure as in the ITPC above), and finally calculated the slope of the t-ITPC at the single-participant level. More details are provided in the respective paragraphs below.

###### 2.2.2.4.1 Time-frequency transform

After preprocessing, single-trial EEG data underwent time-frequency transformation (‘*ft_freqanalysis’*) by means of a wavelet-transform (Cohen, 2014). The bandwidth of interest was centered around the stimulation frequency (+/- 1 Hz, i.e., .54 - 2.54 Hz, thus obtaining a 1.54 Hz center frequency), using a frequency resolution of .2 Hz. The number of fitted cycles was set to 3. The single-trial approach results in ‘induced’ (as compared to ‘evoked’) responses. The output was a complex spectrum; no averaging over channels, trials, or participants was performed at this stage.

###### 2.2.2.4.2 Slope of t-ITPC

As for the ITPC above, t-ITPC was obtained by dividing the complex coefficients of TF-data by their absolute values (thus, normalizing the values to be on the unit circle), averaging, and finally taking the absolute value of the complex mean. Next, we calculated the slope of each t-ITPC time-series by fitting a first-order polynomial (‘*polyfit*’ function in MATLAB;*p*) and then deriving a first-order approximation (p(1)*Time+p(2)). The calculation of the slope was performed on a 5-s-long interval starting from the 3rd tone onset.

##### 2.2.2.5 Statistical comparisons

Before assessing group differences in the t-ITPC, we performed Levene’s test for homogeneity of variance. As the*p*returned significant, we implemented non-parametric group comparisons via a Kruskal-Wallis test. With a significant group effect, we later assessed pairwise group comparisons via permutation testing and with a total of 1000 permutations. This iterative procedure would ultimately assess the p value from the original groups against the p obtained from permutations. Analysis steps and results are reported inSuppl. Table 6.

##### 2.2.2.6 Analyses of oscillatory dynamics

We aimed at assessing the tuning of endogenous delta-band neural oscillatory activity towards the stimulation frequency (*Sf*). A self-sustained endogenous oscillator capable of entrainment should adjust its frequency and phase so to align to external temporal regularities, or rhythms. Such an active process is displayed in tuning dynamics during which the endogenous oscillator accelerates and decelerates to align its period to an external rhythm. Thus, we calculated the instantaneous frequency (IF) and quantified acceleration (Acc) dynamics. While processing the auditory rhythm, oscillations may be fluctuating around the*Sf*with various magnitude and deviating from the*Sf*. We consequently calculated metrics of stability (S) and deviation. Each metric was estimated at the single-participant and -trial level as illustrated inFigure 4A: on top, the time-course of delta-band filtered Hilbert-transformed data; in the middle, IF as the derivative of phase evolution over time, considering the sampling frequency; at the bottom, Acc was calculated as the derivative of IF. The dashed pink line on the IF plot indicates the*Sf*. On the right side, the distribution of IF and Acc in the ‘Pre-’, ‘Post-’, and ‘Dur-(ing)’ the listening periods (color-coded in shades of blues), and pooling across the FC channel cluster. The time-series of IF and Acc were further used to estimate S, the stability of the neural signal. Finally, we obtained the Latency and Deviation (not visualized in the top panel): the first corresponds to the first crossing point of IF through the horizontal*Sf*line; the latter is the standard deviation from the*Sf*. Individual data were then pooled into three groups: HC, CE, and BG patients. Details for each of the metrics are provided in the respective sections below.

**Fig. 4.**
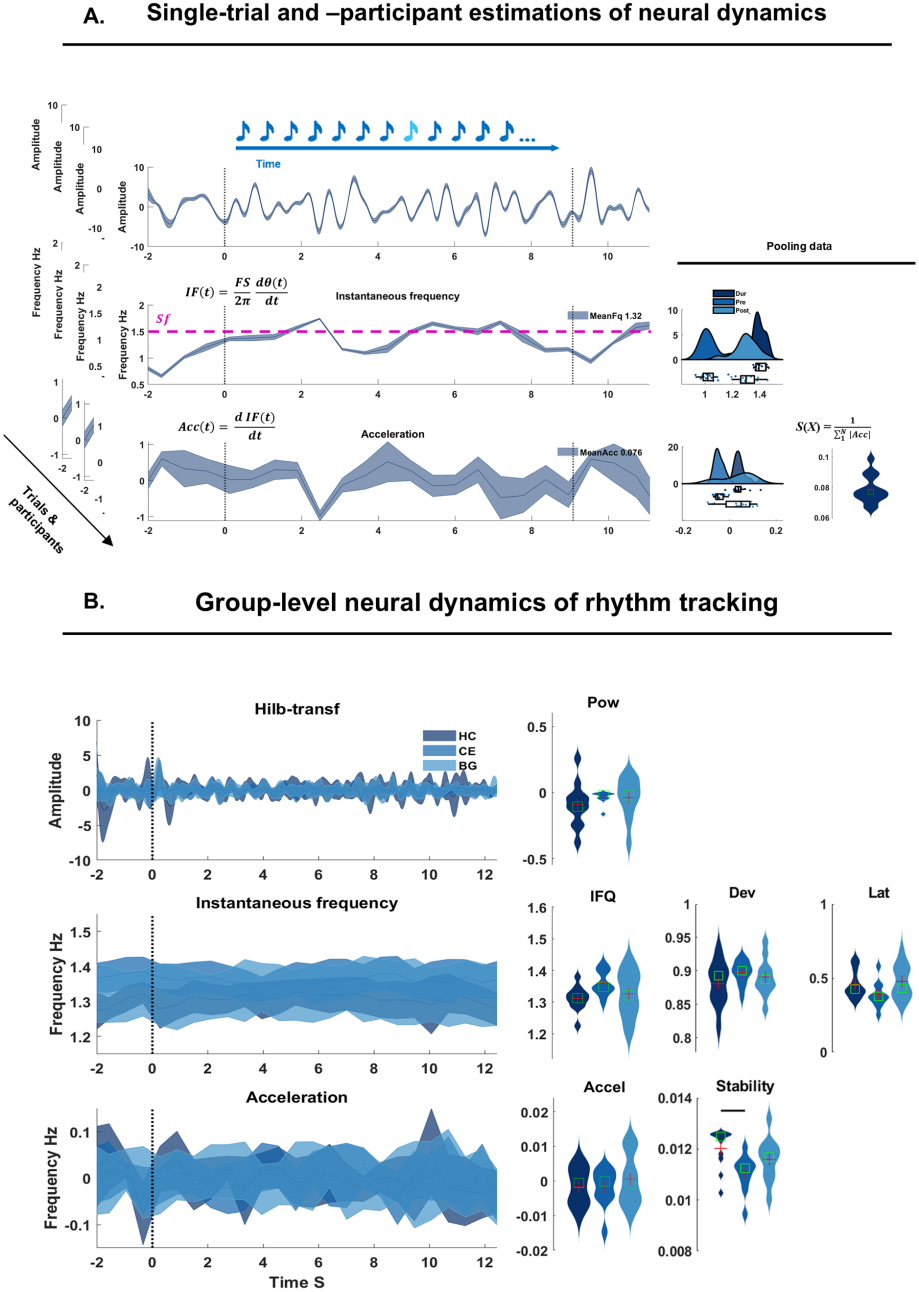
Analyses on neural dynamics of rhythm processing. (A) After obtaining the analytical signal of delta-band activity (top), we estimated, at the single-participant and -trial levels, the instantaneous frequency (IF) and Acceleration (Acc; time-courses on the left side). The pink dashed line on top of the IF indicates the stimulation frequency (Sf). The distribution plots on the right side are obtained by pooling data points over channels and averaging over time. IF and Acc were also used to calculate metrics of Deviation (D) and Stability (S). The violin plots on the right side show the resultant D and S over time, and pooling over channels. (B) mirroring the layout of A, the group-level data (color-coded in shades of blue; solid line reporting the mean across participants; shades represent the standard error across participants) reporting on the left the time-courses of neural oscillations in the delta frequency band (top), the estimated IF (middle) and Acc (bottom). On the right side of the IF time-courses, violin plots of the IF (pooling over participants, but averaging over trials and across the FC cluster of interest). Next to it, the single-participant Dev and Latency. Next to the time-courses of the Acc, violin plots of the Acc (pooling participants and averaging across trials and the FC cluster of interest). On its right, the estimated S values. Red crosses and green squares in violin plots report mean and median respectively. The thick black horizontal lines on top of the violin plots indicate significant group differences.

###### 2.2.2.6.1 Instantaneous frequency

After preprocessing, single-trial EEG data were bandpass-filtered with a 3rd-order Butterworth filter centered around the stimulation frequency (plus and minus 1 Hz, .54–2.54 Hz, obtaining a 1.54 Hz center frequency;*ft_preprocessing*) and Hilbert-transformed to extract the analytic signal. Next, the instantaneous frequency (IF) at each time point (t), and for each channel, trial, and participant, can be calculated with the following formula:

$$IF(t)=\frac{FS}{2\pi }\omega (t)=\frac{FS}{2\pi }\frac{d\theta (t)}{dt}$$

Whereω(t) is the derivative of the unwrapped phase (θ) at each time point (t), given the time-steps (dt) and FS is the sampling frequency. IF can also be obtained in MATLAB using the ‘*instfreq*’ function.

###### 2.2.2.6.2 Acceleration

Once calculated the single-participant, -trial, and channel-level IF, acceleration (Acc) was calculated as the first derivative of IF. Thus, we employed the following formula:

$$Acc(t)=\frac{d\;\;IF(t)}{dt}$$

###### 2.2.2.6.3 Stability

After the single-participant, -trial, and channel-level Acc was obtained, Stability (S) was calculated as the inverse of the sum of absolute changes in Acceleration. Thus, we employed the following formula:

$$S(X)=\frac{1}{\sum_{1}^{N}\left|Acc\right|}$$

###### 2.2.2.6.4 Deviation

Once the single-participant, -trial, and channel-level IF was obtained, we quantified the standard deviation (D) from the stimulation frequency. D was calculated as the square-root of the mean squared difference between the IF and the stimulation frequency (*Sf*):

$$D(X)=\sqrt{\frac{1}{N}\sum_{1}^{N}({\left(IF-Sf\right)}^{2})}$$

###### 2.2.2.6.5 Latency

For each participant, trial, and channel, we estimated how long it took for the IF to tune to the*Sf*(1.5 Hz). The search was performed within the first 8 s of listening (–8 s) of each trial, and by using a narrow frequency criterion (*Sf±*.2 Hz).

###### 2.2.2.6.6 Statistical comparisons

Group differences were statistically assessed for each of the above-mentioned metrics by means of a one-way ANOVA with a group factor (*anova1*built-in into MATLAB), followed by post-hoc simple tests corrected for multiple comparisons (Tukey-Davis correction) (*multcompare*built-in into MATLAB) in case of a significant (*p *< .05) main effect. Simple effects with a*p-*value below an alpha-corrected .05 were considered statistically significant and are depicted as horizontal black lines on top ofFigure 4B. The ANOVA tables and simple tests for significant effects are provided inSuppl. Table 6.

## 3 Results

In this EEG study, we adopted two complementary methodological approaches.

First, we reproduced typical event-related potential (ERP) and inter-trial phase coherence (ITPC) analyses to test*if*and*how*participants’ neural activity encoded the temporal regularity of sound onsets. In the second analysis step, we employed a time-resolved metric of ITPC (t-ITPC) to quantify the build-up of phase-coherence over the course of the auditory sequence. The t-ITPC slope could indicate a mechanism of phase-alignment toward next sound onsets (Breska & Ivry, 2020;Ten Oever et al., 2017). Next, we introduced a new method to assess the tuning of endogenous delta-band neural oscillatory activity toward the*Sf*. We quantified trial-level instantaneous frequency (IF), its dynamics of acceleration while tuning- and de-tuning to the Sf, its Stability (S), and Deviation (Dev) from the Sf.

Details for each methodological approach can be found in the respective methods section above. Results for each method are discussed below.

### 3.1 Event-related potential (ERP) analyses

After computing the sequence-level (Fig. 2B) and tone-level ERPs (Fig. 2C), we obtained single-participant N100 peak amplitude, latency (Fig. 2D, top) and later estimated the variance across tone positions (Fig. 2D, bottom). Participant-level N100 peak amplitude and latency separately entered a Mixed-Effect Model with a Group Factor and a random intercept per participant. The first model reported a significant group effect for N100 peak amplitude (F(2,165) = 2.33,*p*= .02; (Suppl. Table 2). Post-hoc pairwise comparisons showed that CE had a lower N100 amplitude peak than HC (Obs Diff = -3.23,*p*= .01, Eff size = -1.33). There were no significant differences between HC and BG, nor between CE and BG (Suppl. Table 1).

The second model reported no significant group effect for N100 peak amplitude latency (F(2,165) = -.16,*p*= .88). Next, we assessed the variability in the N100 peak amplitude via a one-way ANOVA with a group factor. The ANOVA reported a significant group effect (F(2,30) = 21.26,*p*< .001;Suppl. Table 2, middle), and pairwise post-hoc comparison revealed that HC had higher variance than either CE or BG, but there was no difference between CE and BG (Suppl. Table 2, bottom). Finally, we assessed the variability in N100 peak amplitude latency. The ANOVA reported a significant group effect (F(2,30) = 22.14,*p*< .001;Suppl. Table 3, middle) and pairwise post-hoc comparison revealed that both CE and BG had higher variance than HC but there was no difference between CE and BG (Suppl. Table 3, bottom).

Additional analyses confirmed that there were no group differences in the amplitude and variance of P50, N100, and P200 peaks in the first two tones of the auditory sequence (Suppl. Fig. 1).

### 3.2 Inter-trial phase coherence (ITPC)

While participants listened to isochronous auditory sequences presented at an*Sf*of 1.5 Hz (Fig. 2A), their neural activity responded timely to tone onsets, as shown by the coherence peaks at 1.5 Hz in the ITPC spectrum (Fig. 3A, left). A distinct peak at 1.5 Hz was present in the ITPC spectrum for all groups. However, statistical analyses revealed significant group differences (Fig. 3A, right): HC (dark blue) showed stronger coherence at the*Sf*than both CE and BG groups (lighter shades of blue). The one-way ANOVA revealed a significant group effect (F(2,30) = 5.25,*p*= .01;Suppl. Table 4), and post-hoc simple t-tests revealed higher coherence in HC than in both CE (*p*= .04) and BG (*p=*.02) groups, but no significant difference between CE and BG (Suppl. Table 4; corrected for multiple comparisons via Tukey-Kramer). To confirm the specificity of the effects to neural dynamics centered at the stimulation frequency, we conducted additional statistical analyses on rhythm-unrelated frequency-bands. Following the same statistical procedure detailed above, we showed no group differences in the alpha (8–12 Hz) frequency band, neither for the ITPC nor the Fourier spectrum (Suppl. Fig. 2).

### 3.3 Time-resolved ITPC

We estimated a time-resolved metric of ITPC (t-ITPC) to quantify the build-up of phase-coherence over the course of the auditory sequence. T-ITPC was obtained by quantifying phase coherence from the complex spectra of continuous wavelet transformed data in the delta frequency band. The group-level time course is displayed inFigure 3B, color-coded per group. Next, we calculated, at the level of the single-participant, the slope of t-ITPC over a 5 s interval starting from the third tone onset. Participant data were pooled into groups (Fig. 3B, right), and we later performed group comparisons via non-parametric analysis of variance via a Kruskal-Wallis test. The Group effect was significant (Chi-sq (2,30) = 15.4,*p*< .001), and pairwise group comparisons performed via permutation testing (1000 permutations) revealed a significantly steeper t-ITPC slope for HC than for both CE and BG patients (Suppl. Table 5).

### 3.4 Analyses on neural dynamics

To assess the tuning of endogenous delta-band neural oscillatory activity toward the*Sf*, we quantified trial-level instantaneous frequency (IF), its dynamics of acceleration while tuning- and de-tuning to the*Sf*, its Stability (S), and Deviation (Dev) from the Sf. The group-level data are provided inFigure 4B. On the right side, the violin plots show (in order) the IF and Acc, obtained by averaging data during the listening window (0-8 s) and across channels and trials. Next to it are the distributions of Dev, S, and latency.

We performed independent comparisons for the metrics mentioned above and focused on the listening period only (‘Dur’). The only group comparison showing a significant effect was for Stability: F(2,30) = 4.36,*p*= .02. Post-hoc simple tests showed that CE had lower Stability than HC (*p*= .016;Suppl. Table 6), while no difference was found between HC-BG nor between CE-BG (*p>*.05). The reduced Stability in CE patients reflects high variability in acceleration dynamics of delta-band activity, ultimately suggesting a poorer neural representation of temporal regularity displayed in the auditory sequences. No group difference was detected for Dev nor other metrics (*p>*.05).

## 4 Discussion

In this EEG study, we investigated the causal contributions of the cerebellum (CE) and the basal ganglia (BG) to the capacities to encode and predict temporally regular auditory streams.

We first assessed the encoding of sound onset timing via event-related (ERP) and inter-trial phase coherence (ITPC) analyses. The assessment of the N100 amplitude and latency along with the ITPC amplitude at the*Sf*(1.5 Hz) allowed testing whether the participants’ neural activity responded in time to sound onsets, thus encoding their precise timing. In line with previous evidence, we expected CE but not BG patients to show altered encoding of sound onsets: this would result in increased N100 latency variability and reduced ITPC.

While ITPC and similar phase concentration measures are typically interpreted as a proxy of entrainment, it is generally hard to disentangle true entrainment from a series of evoked responses (Breska & Deouell, 2017;Haegens & Zion Golumbic, 2018;Obleser et al., 2017;Zoefel et al., 2018). In fact, simulations showed that evoked responses drive high-phase coherence (Breska & Deouell, 2017;Obleser et al., 2017), ultimately weakening its functional link with temporal prediction. Thus, in a second analysis step, we introduced a novel approach to assess finer-grained measures of neural dynamics of temporal processing. First, we employed a time-resolved metric of ITPC (t-ITPC) to quantify the build-up of phase-coherence over the course of the auditory sequence. Such a metric was previously shown to be a good indicator of temporal prediction (Breska & Ivry, 2020) and auditory processing (Ten Oever et al., 2017). Next to event-related t-ITPC peaks, which are induced by the phase rest of oscillations at tone onsets, the t-ITPC slope could indicate a mechanism of anticipation of next sound onsets. Thus, we expected BG but not CE patients to show a shallower slope of t-ITPC along the auditory sequence, potentially indicating altered temporal predictions.

Next, we showcased new metrics to assess the tuning of endogenous delta-band neural oscillatory activity toward the*Sf*. We quantified trial-level instantaneous frequency (IF), its dynamics of acceleration while tuning- and de-tuning to the Sf, its Stability (S), and Deviation (Dev) from the*Sf*. We expected BG and CE patients to show reduced S and greater Dev than HC, indicating their altered capacity to align neural dynamics to predictable onsets.

We found that CE patients showed lower N100 amplitudes, and increased N100 latency variability than healthy controls (HC). Furthermore, they displayed a reduced ITPC at the*Sf*, and a nearly flat t-ITPC slope. In contrast, HC showed an increasing slope over the course of the isochronous tone sequence, indicating increased alignment of oscillatory activity to the temporal regularity. Finally, analyses of neural dynamics revealed a tendency toward an increased deviation from the*Sf*, and a significant reduction in stability in CE compared to HC. Thus, while not deviating largely from the*Sf*, reduced stability might reflect abundant fluctuations in acceleration dynamics. Altogether, increased variability in ERPs, reduced delta-band phase coherence, reduced slope of t-ITPC, and reduced stability of delta-band dynamics observed in CE patients suggest altered neural encoding of temporally regular sound onsets and impaired temporal predictions. These observations align with previous evidence linking the CE to the encoding of the precise timing of sensory events (Bareš et al., 2018;Ivry et al., 1988;Ivry & Keele, 1989;Ivry & Schlerf, 2008) and to the processing of temporal intervals (Breska & Ivry, 2020,^2021^;Grube, Cooper, et al., 2010;Grube, Lee, et al., 2010;Teki, Grube, & Griffiths, 2011;Teki, Grube, Kumar, et al., 2011). Thus, these results confirm that CE lesions affect the capacity to predict temporal intervals (Breska & Ivry, 2018,^2020^,^2021^;Grube, Cooper, et al., 2010), potentially impacting the capacity to synchronize ongoing neural activity with external rhythms (Ivry & Keele, 1989;Ivry et al., 1988). However, differently from previous literature (e.g.,Breska & Ivry, 2018,^2020^,^2021^), we show that timing processes in CE lesion patients is not comparable to healthy controls when processing isochronous tone sequences. Possible interpretations for this and other discrepancies related to prior research are provided in the following.

Regarding BG lesion patients, we found increased variability in the N100 peak amplitude latency and a significant reduction in ITPC peak at the*Sf*. Furthermore, BG lesions were associated with a reduced slope of the t-ITPC. No differences were found in the neural dynamics metrics (stability and deviation) which indicates similar tuning dynamics toward the*Sf*(stability) and small deviations from the*Sf*(deviation) in delta-band activity, as observed in HCs. The increased latency variability observed in ERPs and the reduction of the ITPC peak at the*Sf*suggest altered encoding of temporal regularity in auditory sequences (Breska & Ivry, 2020;Nozaradan et al., 2017;Schwartze et al., 2015). The reduction in the t-ITPC slope suggests that BG lesions causally impacted the generation of expected tone onsets, thus aligning with and supporting prior evidence linking the BG with temporal prediction (Grahn, 2009;Grahn & Brett, 2009;Schwartze et al., 2011;Teki, Grube, Kumar, et al., 2011).

While most of the employed metrics (variability in ERPs, ITPC, t-ITPC) did not dissociate specific contributions of BG and CE to sound processing in isochronous sequences, CE but not BG patients clearly displayed reduced ‘stability’. Both ‘stability’ and ‘deviance’ are new metrics to quantify fine-grained fluctuations in delta-band dynamics while tuning- to and de-tuning from the temporal regularity (1.5 Hz; the*Sf*) in isochronous tone sequences. ‘Deviance’ indicates the degree of deviation from the*Sf:*in both patient groups, instantaneous delta-band activity fluctuated around the*Sf*, comparable to HCs. The ‘stability’ metric quantifies fluctuations in acceleratory dynamics while tuning-in and -out of*Sf*. Change in this metric only showed in CE patients, and the reduction in ‘stability’ likely highlights a poorer neural encoding of event onsets in isochronous tone sequences and of temporal intervals. This observation supports the fundamental role of the CE in generating and maintaining stable neural representations of temporal regularities in the sensory environment.

Most of the current results, however, fail to reconcile with previous literature showing fundamental dissociations between rhythm- and interval-based temporal predictions in BG and CE lesion patients (e.g.,Breska & Ivry, 2018;Schwartze & Kotz, 2013), and with evidence showing that temporal predictions in CE lesion patients are comparable to healthy controls when tested in rhythmic contexts (Breska & Ivry, 2018,^2020^,^2021^).

How can these differences be explained? A few factors might have mediated these differences: the experimental paradigm, attention, sample size, and the exclusive focus on delta-band activity. Furthermore, compensatory strategies may further mask results. In fact, in contrast to prior work employing a few repeated temporal intervals and active task settings (e.g.,Breska & Ivry, 2018), here we tested endogenous timing: participants were exposed for 20 min to 8-to-11 s long auditory sequences, and their attention was diverted from the temporal aspects of the sequence as they were asked to count the N of deviant (amplitude attenuated) tones. Thus, the long exposure to an auditory isochronous stream and the absence of an explicit timing task allowed us to test temporal processing in absence of attention. Consequently, we might argue that, at least in passive listening, we cannot dissociate the roles of BG and CE. The use of isochronous auditory sequences, as opposed to complex auditory rhythms, might represent another limitation as these stimuli may have been suboptimal to dissociate CE-related sensory timing computations from BG-related temporal predictions. The small sample size may represent another limitation, as it might have not provided the adequate statistical power to discern such fine dissociations. In addition, it is possible that both patient populations employed compensatory mechanisms, thus masking out existing differences. In fact, we have previously discussed that BG and CE are part of a widespread cortico-subcortical network engaged in rhythm processing. Animal literature shows that there are neurons in the cortex acting as ‘neural chronometer’ encoding the passage of time and the precise timing of sensory events (Merchant et al., 2011;Merchant, Harrington, et al., 2013;Merchant, Pérez, et al., 2013). These cortical neurons may take over timing computations as a functional reorganization mechanism after subcortical brain lesions, ultimately preventing to dissociate BG and CE’s roles in basic timing functions.

Finally, another limitation lies in the difficulty to distinguish fine-grained timing processing in the absence of spatiotemporally resolved data. In this perspective, this research field would benefit from a more accurate characterization of the time-course, strength, and directionality of information flow during rhythm processing. Such an approach would allow to monitor the interplay and the causal relationship between cortico-subcortical regions, as well as between the BG and CE. Furthermore, it would enable to characterize the frequency-specificity and directionality of influence of some timing computations. In fact, while we here focused on delta-band activity only, literature suggests beta-band activity to also be prominent in subcortical and cortical brain regions (Bartolo et al., 2014;Bartolo & Merchant, 2015;Keitel et al., 2017;Keitel & Gross, 2016) and to further be linked to predictive priming of sensory regions (Arnal, 2012;Engel & Fries, 2010). In other words, the anticipatory alignment of beta-band activity to the*when*of salient events (Fujioka et al., 2012,^2015^) couples with bottom-up delta-band activity (Abbasi et al., 2018;Arnal et al., 2015;Merchant et al., 2015;Saleh et al., 2010) to instantiate motor-to-auditory predictions in support of adaptive behavior. Accumulating evidence links cerebellar beta-band activity to temporal and spatial predictions, as well as to the fine processing of the timing of stimuli and action (Andersen & Dalal, 2024). In fact, beta-band activity from the cerebellum was associated with sensory predictions in temporally regular contexts (Andersen & Lundqvist, 2019). Cerebellar beta-band strength is modulated by the temporal predictability of sensory streams (Andersen & Dalal, 2021), and might have a general, modality-independent, role in building temporal expectations during sensory and sensorimotor processes (Herrojo Ruiz et al., 2017). Thus, our focus on scalp activity and on delta-band activity only may have prevented a full characterization of bottom-up and top-down mechanisms of temporal processing and prediction in BG and CE lesion patients. We encourage future studies to better elucidate the link between the observed alterations in delta-band dynamics and top-down beta-band modulatory activity.

Overall, the newly employed metrics allowed characterizing dynamics of delta-band oscillations in basic rhythm processing: we showed that delta oscillations fluctuate over time,*tuning in*and*out of*perceived acoustic regularity in auditory sequences by dynamically accelerating and slowing down from silence to listening periods, and further increasing phase coherence over time. These observations support the*dynamic attending*hypothesis (Jones & Boltz, 1989;Large & Jones, 1999), and confirm the role of low-frequency oscillations (Schroeder & Lakatos, 2009) in encoding and predicting temporal regularities in the auditory environment. On the other hand, the current approach also confirmed the fundamental role of the BG and CE in an extended cortico-subcortical-cortical network underlying rhythm and timing processing (Criscuolo, Pando-Naude, et al., 2022;Kasdan et al., 2022;Kotz et al., 2018;Schwartze & Kotz, 2013).

In fact, we showed that while the healthy brain could flexibly and dynamically respond to and synchronize with sensory inputs, patients with lesions in the BG and CE did not. Patients showed a degree of heterogeneity and deteriorated capacity to synchronize ongoing neural activity to temporal regularities in the acoustic environment. Differently from our hypotheses, however, our EEG data did not clearly dissociate CE from BG timing functions in isochronous contexts. While we delineate potential limitations in our experimental paradigm, these observations motivate novel research on the causal role of subcortical circuitries in basic timing processing. We encourage future studies to complement the current findings with spatiotemporally resolved assessments of delta-, beta-band activity and their dynamic interaction. Such a complementary approach would deepen our understanding of the neurophysiological mechanisms underlying intra- and inter-individual variabilities in the capacities to detect, produce and synchronize with temporal regularities in the sensory environment, and to ultimately produce adaptive behavior.

## 5 Conclusions

The capacities to encode the precise timing of the sensory events around us, and to time our (re-) actions to changes in the environment are pivotal to act and adapt in a dynamically changing environment. In this study, we explored the rich and variegated landscape of neural oscillatory dynamics, and assessed*if, when*, and*how*neural oscillations processed the temporal regularity in acoustic sequences. BG and CE lesions causally impacted the neurophysiological encoding of the rhythm, as demonstrated by reduced capacities to encode precise tone onsets and predict the timing next events.

## Supplementary Material

Supplementary Material

## Data Availability

The data and analysis code in use will be made available upon reasonable request by the corresponding author.

## References

[b1] Abbasi , O. , Hirschmann , J. , Storzer , L. , Özkurt , T. E. , Elben , S. , Vesper , J. , Wojtecki , L. , Schmitz , G. , Schnitzler , A. , & Butz , M . ( 2018 ). Unilateral deep brain stimulation suppresses alpha and beta oscillations in sensorimotor cortices . NeuroImage , 174 , 201 – 207 . 10.1016/j.neuroimage.2018.03.026 29551459

[b2] Andersen , L. M. , & Dalal , S. S . ( 2021 ). The cerebellar clock: Predicting and timing somatosensory touch . NeuroImage , 238 , 118202 . 10.1016/J.NEUROIMAGE.2021.118202 34089874

[b3] Andersen , L. M. , & Dalal , S. S . ( 2024 ). The role of the cerebellum in timing . Current Opinion in Behavioral Sciences , 59 , 101427 . 10.1016/J.COBEHA.2024.101427

[b4] Andersen , L. M. , & Lundqvist , D . ( 2019 ). Somatosensory responses to nothing: An MEG study of expectations during omission of tactile stimulations . NeuroImage , 184 , 78 – 89 . 10.1016/J.NEUROIMAGE.2018.09.014 30213774

[b5] Arnal , L. H . ( 2012 ). Predicting “When” using the motor system’s beta-band oscillations . Frontiers in Human Neuroscience , 6 , 225 . 10.3389/fnhum.2012.00225 22876228 PMC3410664

[b6] Arnal , L. H. , Doelling , K. B. , & Poeppel , D . ( 2015 ). Delta-beta coupled oscillations underlie temporal prediction accuracy . Cerebral Cortex , 25 ( 9 ), 3077 – 3085 . 10.1093/cercor/bhu103 24846147 PMC4537446

[b7] Arnal , L. H. , & Giraud , A. L . ( 2012 ). Cortical oscillations and sensory predictions . Trends in Cognitive Sciences , 16 ( 7 ), 390 – 398 . 10.1016/j.tics.2012.05.003 22682813

[b8] Bareš , M. , Apps , R. , Avanzino , L. , Breska , A. , D’Angelo , E. , Filip , P. , Gerwig , M. , Ivry , R. B. , Lawrenson , C. L. , Louis , E. D. , Lusk , N. A. , Manto , M. , Meck , W. H. , Mitoma , H. , & Petter , E. A . ( 2018 ). Consensus paper: Decoding the contributions of the cerebellum as a time machine. From neurons to clinical applications . The Cerebellum , 18 ( 2 ), 266 – 286 . 10.1007/S12311-018-0979-5 30259343

[b9] Bartolo , R. , & Merchant , H . ( 2015 ). β oscillations are linked to the initiation of sensory-cued movement sequences and the internal guidance of regular tapping in the monkey . Journal of Neuroscience , 35 ( 11 ), 4635 – 4640 . 10.1523/JNEUROSCI.4570-14.2015 25788680 PMC6605135

[b10] Bartolo , R. , Prado , L. , & Merchant , H . ( 2014 ). Information processing in the primate basal ganglia during sensory-guided and internally driven rhythmic tapping . Journal of Neuroscience , 34 ( 11 ), 3910 – 3923 . 10.1523/JNEUROSCI.2679-13.2014 24623769 PMC6705277

[b11] Breska , A. , & Deouell , L. Y . ( 2017 ). Neural mechanisms of rhythm-based temporal prediction: Delta phase-locking reflects temporal predictability but not rhythmic entrainment . PLoS Biology , 15 ( 2 ), e2001665 . 10.1371/journal.pbio.2001665 28187128 PMC5302287

[b12] Breska , A. , & Ivry , R. B . ( 2018 ). Double dissociation of single-interval and rhythmic temporal prediction in cerebellar degeneration and Parkinson’s disease . Proceedings of the National Academy of Sciences of the United States of America , 115 ( 48 ), 12283 – 12288 . 10.1073/PNAS.1810596115 30425170 PMC6275527

[b13] Breska , A. , & Ivry , R. B . ( 2020 ). Context-specific control over the neural dynamics of temporal attention by the human cerebellum . Science Advances , 6 ( 49 ), eabb1141 . 10.1126/SCIADV.ABB1141 33268365 PMC7821877

[b14] Breska , A. , & Ivry , R. B . ( 2021 ). The human cerebellum is essential for modulating perceptual sensitivity based on temporal expectations . ELife , 10 , e66743 . 10.7554/ELIFE.66743 34165079 PMC8245126

[b15] Cohen , M. X . ( 2014 ). Analyzing neural time series data: Theory and practice . MIT Press . 10.7551/mitpress/9609.001.0001

[b16] Cravo , A. M. , Rohenkohl , G. , Wyart , V. , & Nobre , A. C . ( 2013 ). Temporal expectation enhances contrast sensitivity by phase entrainment of low-frequency oscillations in visual cortex . Journal of Neuroscience , 33 ( 9 ), 4002 – 4010 . 10.1523/JNEUROSCI.4675-12.2013 23447609 PMC3638366

[b17] Criscuolo , A. , Pando-Naude , V. , Bonetti , L. , Vuust , P. , & Brattico , E . ( 2022 ). An ALE meta-analytic review of musical expertise . Scientific Reports , 12 , 11726 . 10.1038/s41598-022-14959-4 35821035 PMC9276732

[b18] Criscuolo , A. , Schwartze , M. , Bonetti , L. , & Kotz , S. A . ( 2024 ). Aging impacts basic auditory and timing processes . BioRxiv , 2024.03.24.586049. 10.1101/2024.03.24.586049 PMC11874193 40026217

[b19] Criscuolo , A. , Schwartze , M. , Henry , M. J. , Obermeier , C. , & Kotz , S. A . ( 2023 ). Individual neurophysiological signatures of spontaneous rhythm processing . NeuroImage , 273 , 120090 . 10.1016/j.neuroimage.2023.120090 37028735

[b20] Criscuolo , A. , Schwartze , M. , & Kotz , S. A . ( 2022 ). Cognition through the lens of a body–brain dynamic system . Trends in Neurosciences , 45 ( 9 ), 667 – 677 . 10.1016/J.TINS.2022.06.004 35810022

[b21] Criscuolo , A. , Schwartze , M. , Prado , L. , Ayala , Y. , Merchant , H. , & Kotz , S. A . ( 2023 ). Macaque monkeys and humans sample temporal regularities in the acoustic environment . Progress in Neurobiology , 229 , 102502 . 10.1016/J.PNEUROBIO.2023.102502 37442410

[b22] Engel , A. K. , & Fries , P . ( 2010 ). Beta-band oscillations-signalling the status quo? Current Opinion in Neurobiology , 20 ( 2 ), 156 – 165 . 10.1016/j.conb.2010.02.015 20359884

[b71] Fraisse , P. ( 1963 ). The psychology of time . Harper & Row . https://psycnet.apa.org/record/1965-00055-000

[b23] Friston , K . ( 2005 ). A theory of cortical responses . Philosophical Transactions of the Royal Society B: Biological Sciences , 360 ( 1456 ), 815 – 836 . 10.1098/rstb.2005.1622 PMC1569488 15937014

[b24] Fujioka , T. , Ross , B. , & Trainor , L. J . ( 2015 ). Beta-band oscillations represent auditory beat and its metrical hierarchy in perception and imagery . Journal of Neuroscience , 35 ( 45 ), 15187 – 15198 . 10.1523/JNEUROSCI.2397-15.2015 26558788 PMC6605356

[b25] Fujioka , T. , Trainor , L. J. , Large , E. W. , & Ross , B . ( 2009 ). Beta and gamma rhythms in human auditory cortex during musical beat processing . Annals of the New York Academy of Sciences , 1169 , 89 – 92 . 10.1111/j.1749-6632.2009.04779.x 19673759

[b26] Fujioka , T. , Trainor , L. J. , Large , E. W. , & Ross , B . ( 2012 ). Internalized timing of isochronous sounds is represented in neuromagnetic beta oscillations . Journal of Neuroscience , 32 ( 5 ), 1791 – 1802 . 10.1523/JNEUROSCI.4107-11.2012 22302818 PMC6703342

[b27] Gámez , J. , Yc , K. , Ayala , Y. A. , Dotov , D. , Prado , L. , & Merchant , H . ( 2018 ). Predictive rhythmic tapping to isochronous and tempo changing metronomes in the nonhuman primate . Annals of the New York Academy of Sciences . 10.1111/nyas.13671 29707785

[b28] Grahn , J. A . ( 2009 ). The role of the basal ganglia in beat perception . Annals of the New York Academy of Sciences , 1169 ( 1 ), 35 – 45 . 10.1111/j.1749-6632.2009.04553.x 19673753

[b29] Grahn , J. A. , & Brett , M . ( 2009 ). Impairment of beat-based rhythm discrimination in Parkinson’s disease . Cortex , 45 ( 1 ), 54 – 61 . 10.1016/j.cortex.2008.01.005 19027895

[b30] Greenfield , M. D. , Aihara , I. , Amichay , G. , Anichini , M. , & Nityananda , V . ( 2021 ). Rhythm interaction in animal groups: selective attention in communication networks . Philosophical Transactions of the Royal Society B , 376 ( 1835 ). 10.1098/RSTB.2020.0338 PMC8387861 34420386

[b31] Greenfield , M. D. , Honing , H. , Kotz , S. A. , & Ravignani , A . ( 2021 ). Synchrony and rhythm interaction: From the brain to behavioural ecology . Philosophical Transactions of the Royal Society B: Biological Sciences , 376 ( 1835 ). 10.1098/rstb.2020.0324 PMC8384058 34420379

[b32] Grube , M. , Cooper , F. E. , Chinnery , P. F. , & Griffiths , T. D . ( 2010 ). Dissociation of duration-based and beat-based auditory timing in cerebellar degeneration . Proceedings of the National Academy of Sciences of the United States of America , 107 ( 25 ), 11597 – 11601 . 10.1073/pnas.0910473107 20534501 PMC2895141

[b33] Grube , M. , Lee , K. H. , Griffiths , T. D. , Barker , A. T. , & Woodruff , P. W . ( 2010 ). Transcranial magnetic theta-burst stimulation of the human cerebellum distinguishes absolute, duration-based from relative, beat-based perception of subsecond time intervals . Frontiers in Psychology , 1 , 1946 . 10.3389/fpsyg.2010.00171 PMC3153783 21833234

[b34] Haegens , S. , & Zion Golumbic , E . ( 2018 ). Rhythmic facilitation of sensory processing: A critical review . Neuroscience and Biobehavioral Reviews , 86 , 150 – 165 . 10.1016/j.neubiorev.2017.12.002 29223770

[b35] Herrojo Ruiz , M. , Maess , B. , Altenmüller , E. , Curio , G. , & Nikulin , V. V . ( 2017 ). Cingulate and cerebellar beta oscillations are engaged in the acquisition of auditory-motor sequences . Human Brain Mapping , 38 ( 10 ), 5161 – 5179 . 10.1002/HBM.23722 28703919 PMC6866917

[b36] Ivry , R. B. , & Keele , S. W . ( 1989 ). Timing functions of the cerebellum . Journal of Cognitive Neuroscience , 1 ( 2 ), 136 – 152 . 10.1162/JOCN.1989.1.2.136 23968462

[b37] Ivry , R. B. , Keele , S. W. , & Diener , H. C . ( 1988 ). Dissociation of the lateral and medial cerebellum in movement timing and movement execution . Experimental Brain Research , 73 (1) , 167 – 180 . 10.1007/BF00279670 3208855

[b38] Ivry , R. B. , & Schlerf , J. E . ( 2008 ). Dedicated and intrinsic models of time perception . Trends in Cognitive Sciences , 12 ( 7 ), 273 – 280 . 10.1016/J.TICS.2008.04.002 18539519 PMC4335014

[b39] Jones , M. R. , & Boltz , M . ( 1989 ). Dynamic attending and responses to time . Psychological Review , 96 ( 3 ), 459 – 491 . 10.1037/0033-295X.96.3.459 2756068

[b40] Kasdan , A. V. , Burgess , A. N. , Pizzagalli , F. , Scartozzi , A. , Chern , A. , Kotz , S. A. , Wilson , S. M. , & Gordon , R. L . ( 2022 ). Identifying a brain network for musical rhythm: A functional neuroimaging meta-analysis and systematic review . Neuroscience & Biobehavioral Reviews , 136 , 104588 . 10.1016/J.NEUBIOREV.2022.104588 35259422 PMC9195154

[b41] Keitel , A. , & Gross , J . ( 2016 ). Individual human brain areas can be identified from their characteristic spectral activation fingerprints . PLoS Biology , 14 ( 6 ), 1 – 22 . 10.1371/journal.pbio.1002498 PMC4927181 27355236

[b42] Keitel , A. , Ince , R. A. A. , Gross , J. , & Kayser , C . ( 2017 ). NeuroImage auditory cortical delta-entrainment interacts with oscillatory power in multiple fronto-parietal networks . NeuroImage , 147 , 32 – 42 . 10.1016/j.neuroimage.2016.11.062 27903440 PMC5315055

[b43] Kotz , S. A. , Ravignani , A. , & Fitch , W. T . ( 2018 ). The evolution of rhythm processing . Trends in Cognitive Sciences , 22 ( 10 ), 896 – 910 . 10.1016/j.tics.2018.08.002 30266149

[b44] Kotz , S. A. , & Schmidt-Kassow , M . ( 2015 ). Basal ganglia contribution to rule expectancy and temporal predictability in speech . Cortex , 68 , 48 – 60 . 10.1016/j.cortex.2015.02.021 25863903

[b45] Lakatos , P. , Karmos , G. , Mehta , A. D. , Ulbert , I. , & Schroeder , C. E . ( 2008 ). Entrainment of neuronal oscillations as a mechanism of attentional selection . Science , 320 ( 5872 ), 110 – 113 . 10.1126/science.1154735 18388295

[b46] Lakatos , P. , Musacchia , G. , O’Connel , M. N. , Falchier , A. Y. , Javitt , D. C. , & Schroeder , C. E . ( 2013 ). The spectrotemporal filter mechanism of auditory selective attention . Neuron , 77 ( 4 ), 750 – 761 . 10.1016/j.neuron.2012.11.034 23439126 PMC3583016

[b47] Lakatos , P. , Shah , A. S. , Knuth , K. H. , Ulbert , I. , Karmos , G. , & Schroeder , C. E . ( 2005 ). An oscillatory hierarchy controlling neuronal excitability and stimulus processing in the auditory cortex an oscillatory hierarchy controlling neuronal excitability and stimulus processing in the auditory cortex . Journal of Neurophysiology , 94 , 1904 – 1911 . 10.1152/jn.00263.2005 15901760

[b48] Large , E. W. , & Jones , M. R . ( 1999 ). The dynamics of attending: How people track time-varying events . Psychological Review , 106 ( 1 ), 119 – 159 . 10.1037/0033-295X.106.1.119

[b49] Merchant , H. , Grahn , J. , Trainor , L. , Rohrmeier , M. , & Fitch , W. T . ( 2015 ). Finding the beat: A neural perspective across humans and non-human primates . Philosophical Transactions of the Royal Society B: Biological Sciences , 370 , 20140093 . 10.1098/rstb.2014.0093 PMC4321134 25646516

[b50] Merchant , H. , Harrington , D. L. , & Meck , W. H . ( 2013 ). Neural basis of the perception and estimation of time . Annual Review of Neuroscience , 36 , 313 – 336 . 10.1146/annurev-neuro-062012-170349 23725000

[b51] Merchant , H. , Pérez , O. , Zarco , W. , & Gámez , J . ( 2013 ). Interval tuning in the primate medial premotor cortex as a general timing mechanism . Journal of Neuroscience , 33 ( 21 ), 9082 – 9096 . 10.1523/JNEUROSCI.5513-12.2013 23699519 PMC6705035

[b52] Merchant , H. , Zarco , W. , Pérez , O. , Prado , L. , & Bartolo , R . ( 2011 ). Measuring time with different neural chronometers during a synchronization-continuation task . Proceedings of the National Academy of Sciences of the United States of America , 108 ( 49 ), 19784 – 19789 . 10.1073/pnas.1112933108 22106292 PMC3241773

[b53] Morillon , B. , Schroeder , C. E. , Wyart , V. , & Arnal , L. H . ( 2016 ). Temporal prediction in lieu of periodic stimulation . Journal of Neuroscience , 36 ( 8 ), 2342 – 2347 . 10.1523/JNEUROSCI.0836-15.2016 26911682 PMC4860448

[b54] Nobre , A. C. , Rohenkohl , G. , & Stokes , M . ( 2012 ). Nervous anticipation: Top-down biasing across space and time . In M. I. Posner (Ed.), Cognitive neuroscience of attention ( 2nd ed.), pp. 159 – 186 . The Guilford Press . https://psycnet.apa.org/record/2012-02692-012

[b72] Nozaradan , S. , Peretz , I. , & Mouraux , A. ( 2012 ). Selective neuronal entrainment to the beat and meter embedded in a musical rhythm . Journal of Neuroscience , 32 ( 49 ), 17572 – 17581 . 10.1523/JNEUROSCI.3203-12.2012 23223281 PMC6621650

[b55] Nozaradan , S. , Schwartze , M. , Obermeier , C. , & Kotz , S. A . ( 2017 ). Specific contributions of basal ganglia and cerebellum to the neural tracking of rhythm . Cortex , 95 , 156 – 168 . 10.1016/j.cortex.2017.08.015 28910668

[b56] Obleser , J. , Henry , M. J. , & Lakatos , P . ( 2017 ). What do we talk about when we talk about rhythm? PLoS Biology , 15 ( 9 ), e2002794 . 10.1371/journal.pbio.2002794 28926570 PMC5604933

[b57] Oostenveld , R. , Fries , P. , Maris , E. , & Schoffelen , J. M . ( 2011 ). FieldTrip: Open source software for advanced analysis of MEG, EEG, and invasive electrophysiological data . Computational Intelligence and Neuroscience , 2011 , 156869 . 10.1155/2011/156869 21253357 PMC3021840

[b58] Ross , J. , Iversen , J. , & Balasubramaniam , R . ( 2018 ). Dorsal premotor contributions to auditory rhythm perception: Causal transcranial magnetic stimulation studies of interval, tempo, and phase . bioRxiv . 10.1101/368597

[b59] Saleh , M. , Reimer , J. , Penn , R. , Ojakangas , C. L. , & Hatsopoulos , N. G . ( 2010 ). Fast and slow oscillations in human primary motor cortex predict oncoming behaviorally relevant cues . Neuron , 65 ( 4 ), 461 – 471 . 10.1016/j.neuron.2010.02.001 20188651 PMC3199221

[b60] Schroeder , C. E. , & Lakatos , P . ( 2009 ). Low-frequency neuronal oscillations as instruments of sensory selection . Trends in Neurosciences , 32 ( 1 ), 9 – 18 . 10.1016/j.tins.2008.09.012 19012975 PMC2990947

[b61] Schwartze , M. , Keller , P. E. , Patel , A. D. , & Kotz , S. A . ( 2011 ). The impact of basal ganglia lesions on sensorimotor synchronization, spontaneous motor tempo, and the detection of tempo changes . Behavioural Brain Research , 216 ( 2 ), 685 – 691 . 10.1016/j.bbr.2010.09.015 20883725

[b62] Schwartze , M. , & Kotz , S. A . ( 2013 ). A dual-pathway neural architecture for specific temporal prediction . Neuroscience and Biobehavioral Reviews , 37 ( 10 ), 2587 – 2596 . 10.1016/j.neubiorev.2013.08.005 23994272

[b63] Schwartze , M. , & Kotz , S. A . ( 2021 ). Delayed auditory encoding and variable representation of stimulus regularity in cerebellar lesion patients . BioRxiv , 2021.02.06.430035. 10.1101/2021.02.06.430035

[b64] Schwartze , M. , Stockert , A. , & Kotz , S. A . ( 2015 ). Striatal contributions to sensory timing: Voxel-based lesion mapping of electrophysiological markers . Cortex , 71 , 332 – 340 . 10.1016/j.cortex.2015.07.016 26298502

[b65] Snyder , J. S. , & Large , E. W . ( 2005 ). Gamma-band activity reflects the metric structure of rhythmic tone sequences . Cognitive Brain Research , 24 ( 1 ), 117 – 126 . 10.1016/j.cogbrainres.2004.12.014 15922164

[b66] Teki , S. , Grube , M. , & Griffiths , T. D . ( 2011 ). A unified model of time perception accounts for duration-based and beat-based timing . Frontiers in Integrative Neuroscience , 5 , 20319 . 10.3389/FNINT.2011.00090 PMC3249611 22319477

[b67] Teki , S. , Grube , M. , Kumar , S. , & Griffiths , T. D . ( 2011 ). Distinct neural substrates of duration-based and beat-based auditory timing . Journal of Neuroscience , 31 ( 10 ), 3805 – 3812 . 10.1523/JNEUROSCI.5561-10.2011 21389235 PMC3074096

[b68] Ten Oever , S. , Schroeder , C. E. , Poeppel , D. , Van Atteveldt , N. , Mehta , A. D. , Mégevand , P. , Groppe , D. M. , & Zion-Golumbic , E . ( 2017 ). Low-frequency cortical oscillations entrain to subthreshold rhythmic auditory stimuli . Journal of Neuroscience , 37 ( 19 ), 4903 – 4912 . 10.1523/JNEUROSCI.3658-16.2017 28411273 PMC5426181

[b69] Zoefel , B. , ten Oever , S. , & Sack , A. T . ( 2018 ). The involvement of endogenous neural oscillations in the processing of rhythmic input: More than a regular repetition of evoked neural responses . Frontiers in Neuroscience , 12 , 1 – 13 . 10.3389/fnins.2018.00095 29563860 PMC5845906

