# Supplementary material for: Basal ganglia and cerebellar lesions causally impact the neural encoding of temporal regularities
Source: Imaging Neurosci (Camb). 2025 Feb 27;3:imag_a_00492. doi: 10.1162/imag_a_00492 (PMC12319801; doi:10.1162/imag_a_00492)
Supplement: Supplementary Material [file imag_a_00492-supp.pdf]

Supplementary materials

Figures

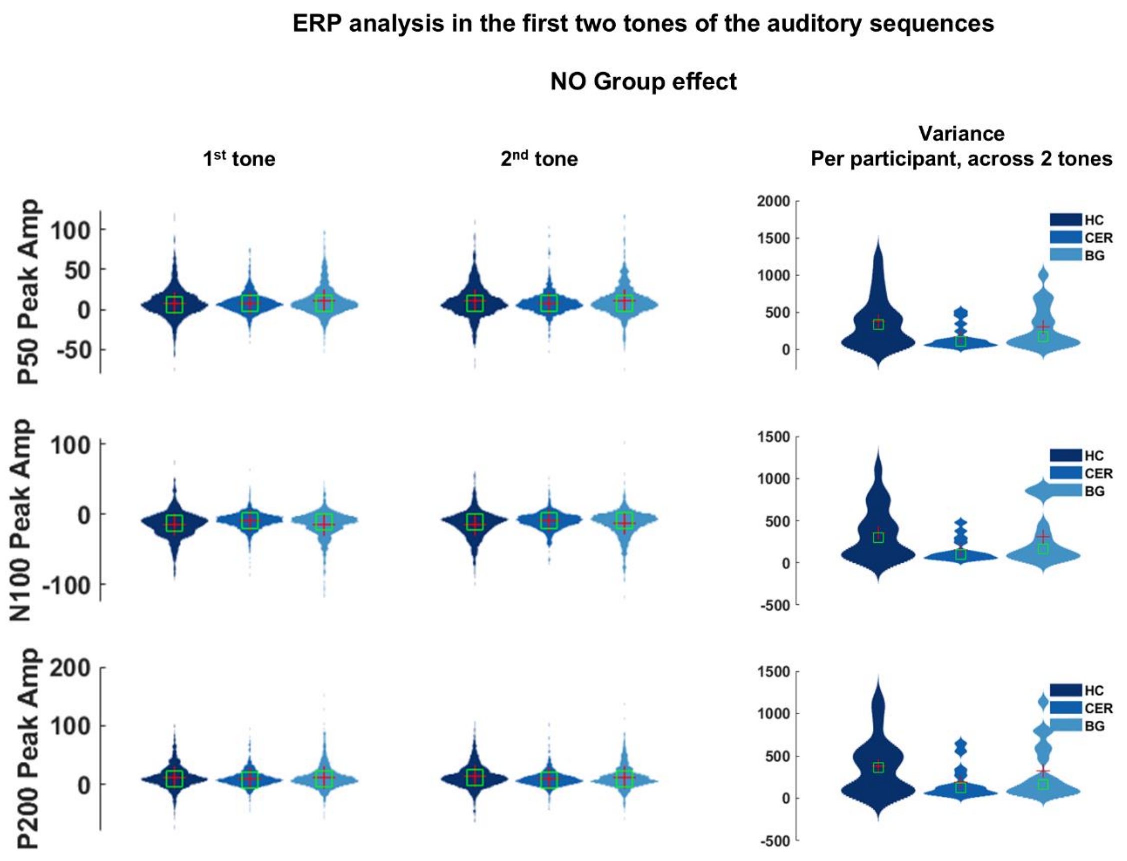

Suppl. Figure 1 – ERP peak analyses in the first two tones of the auditory sequences.
From top to bottom, we provide ERP peak amplitudes for the P50, N100 and P200 components, for the first (1st column) and second (2nd column) tone in the auditory sequence, color coded per group: dark blue for HC, lighter blue for the CE group and light blue for the BG group. In the third column, the ERP peak amplitude variance across the two tone positions.

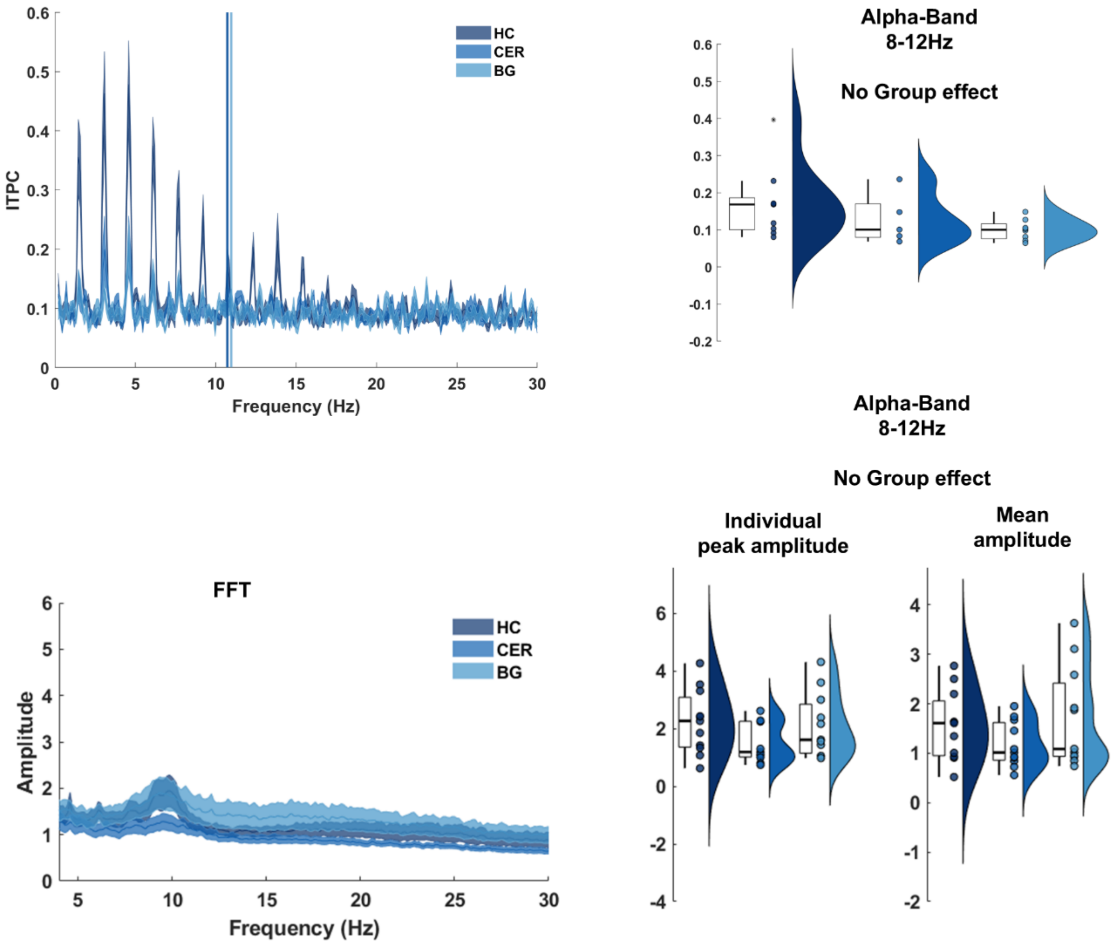

Suppl. Figure 2 – ITPC and FFT analyses on other frequency bands.
In the top row, we provide the inter-trial phase coherence (coherence values on the y-axis) calculated from .5 to 30Hz (frequency on the x-axis). On the right, the coherence values in the alpha-band (8-12Hz) color coded per group. At the bottom, the Fourier (FFT) spectrum (amplitude on the y-axis) ranging from 4 to 30Hz (frequency in the x-axis). On the right, individual peak amplitude and mean amplitude (in order) in the alpha-band (8-12Hz), color coded per participant. As for the plot above, each dot corresponds to one participant's value; the box plot shows the standard error of the mean; the horizontal black line shows the median; the half violins show the distribution of individual data.

Tables

Mixed effect model: ERP N100 peak amplitude

**Model information:**	
**Number of observations**	165
**Fixed effects coefficients**	2
**Random effects coefficients**	33
**Covariance parameters**	2

**Formula:**	varOI ~ 1+ Group + (1 | Participant)

Model fit statistics

AIC	BIC	Log Likelihood	Deviance
782.18	294	-387	774.18

Fixed effects coefficients (95% CI):

	Estimate	SE	tStat	DF	pValue	Lower	Upper
**Intercept**	-5.05	1.23	-4.10	163	6.62e-5	-7.49	-2.62
**Group**	1.33	.57	2.33	163	.02	.2	2.5

Random effects covariance parameters (95% CIs)

	Type	Estimate	Lower	Upper
**Intercept | Participant**	Std	2.52	1.92	3.31
**Residual Std**	Std	2.04	1.81	2.30

Post-hoc comparisons via permutation testing

	pValue	Observed Diff	Effect size
HC – CE	.01	-3.23	-1.33
HC - BG	.06	-2.66	-.87
CE - BG	.43	.56	.39

Suppl. Table 1 – Mixed effect model on ERP N100 Peak Amplitude

The table reports model information: number of observations, fixed effect coefficients, random effect coefficients, covariance parameters. Then, the formula used to fit the model and model fit statistics: AIC, BIC values, Log Likelihood and Deviance. Further below, the fixed effect coefficients in a 95% confidence interval (CI): estimate, standard error, t-stat, degrees of freedom (DF), p value, lower and upper bound. Right below, random effects covariance parameters: estimate, lower and upper bound. At the bottom, the results of post-hoc comparisons performed via permutation testing: p-value, observed difference and effect size.

ERP N100 peak amplitude variance

Levene's test for homogeneity of variance

	F stat	DF	pValue
**Group**	3.73	2, 30	.06

Group Comparison via parametric test: ANOVA

	**SS**	**DF**	**MS**	**F stat**	**pValue**
**Group**	1090	2	545	21.26	<.001
**Error**	308	30	26		

Post-hoc comparisons with Tukey-Kramer correction

	**pValue**	**Observed Diff**	**Lower Limit**	**Upper Limit**
HC – CE	9.2e-5	20.54	12	29
HC - BG	.003	13.53	4.99	22
CE - BG	.11	-7	-15.6	1.54

Suppl. Table 2 – Group comparison for the N100 peak amplitude variance.
In order, the table reports results from the Levene's test for homogeneity of variance: F-statistics, degrees of freedom (DF) and p value. In the middle, the results from the ANOVA: sum of squares (SS), degrees of freedom (DF), mean squared error (MS), F-statistics and p-value. The error term reports the within-group variation. At the bottom, the results of the pairwise group comparison, with Tukey-Kramer correction. In order, the p-value, observed difference, lower and upper limit.

ERP N100 peak amplitude latency variance

Levene's test for homogeneity of variance

	F stat	DF	pValue
**Group**	.21	2, 30	.81

Group Comparison via parametric test: ANOVA

	**SS**	**DF**	**MS**	**F stat**	**pValue**
**Group**	29.47	2	14.73	22.14	9.39e-5
**Error**	7.99	30	.67		

Post-hoc comparisons with Tukey-Kramer correction

	**pValue**	**Observed Diff**	**Lower Limit**	**Upper Limit**
HC – CE	9.34e-5	-3.30	-4.68	-1.93
HC - BG	.001	-2.47	-3.84	-1.09
CE - BG	.28	.84	-.54	2.21

Suppl. Table 3 - Group comparison for the N100 peak amplitude latency variance.
In order, the table reports results from the Levene's test for homogeneity of variance: F-statistics, degrees of freedom (DF) and p value. In the middle, the results from the ANOVA: sum of squares (SS), degrees of freedom (DF), mean squared error (MS), F-statistics and p-value. The error term reports the within-group variation. At the bottom, the results of the pairwise group comparison, with Tukey-Kramer correction. In order, the p-value, observed difference, lower and upper limit.

ITPC at the ***Sf***

**Levene's test for homogeneity of variance**			
	F stat	DF	pValue
**Group**	3.22	2, 30	.06

Group Comparison via parametric test: ANOVA

	**SS**	**DF**	**MS**	**F stat**	**pValue**
**Group**	.25	2	.13	5.25	.01
**Error**	.45	30	.02		

Post-hoc comparisons with Tukey-Kramer correction

	**pValue**	**Observed Diff**	**Lower Limit**	**Upper Limit**
HC – CE	.04	.22	.001	.44
HC - BG	.02	.22	.03	.41
CE - BG	.99	-.001	-.22	.22

Suppl. Table 4 – Group comparison of the ITPC at the stimulation frequency.
In order, the table reports results from the Levene's test for homogeneity of variance: F-statistics, degrees of freedom (DF) and p value. In the middle, the results from the ANOVA: sum of squares (SS), degrees of freedom (DF), mean squared error (MS), F-statistics and p-value. The error term reports the within-group variation. At the bottom, the results of the pairwise group comparison, with Tukey-Kramer correction. In order, the p-value, observed difference, lower and upper limit.

t-ITPC Slope

Levene's test for homogeneity of variance

	F stat	DF	pValue
**Group**	3.34	2, 30	.048

Group Comparison via non-parametric test: Kruskal-Wallis

	**SS**	**DF**	**MS**	**Chi-sq**	**pValue**
**Group**	1440	2	720	15.4	<.001
**Error**	1551	30	52		

Post-hoc comparisons with permutation testing

	**pValue**	**Observed Diff**	**Effect size**
HC – CE	.002	.07	1.66
HC - BG	.01	.06	1.19
CE - BG	.19	-.02	-.60

Suppl. Table 5 – Group comparison of the t-ITPC Slope

In order, the table reports results from the Levene's test for homogeneity of variance: F-statistics, degrees of freedom (DF) and p value. In the middle, the results from the Kruskal-Wallis test: sum of squares (SS), degrees of freedom (DF), mean squared error (MS), Chi-squared and p-value. The error term reports the within-group variation. At the bottom, the results of the pairwise group comparison performed via 1000 permutation tests. In order, the p-value, observed difference, effect size.

Stability of IF

Levene's test for homogeneity of variance

	F stat	DF	pValue
**Group**	.16	2, 30	.85

Group Comparison via parametric test: ANOVA

	**SS**	**DF**	**MS**	**F stat**	**pValue**
**Group**	5.34e-10	2	2.67e-10	4.36	.02
**Error**	1.84e-9	30	6.12e-11		

Post-hoc comparisons with Tukey-Kramer correction

	**pValue**	**Observed Diff**	**Lower Limit**	**Upper Limit**
HC – CE	.016	9.84e-6	1.62e-6	1.81e-5
HC - BG	.27	4.55e-6	-3.67e-6	1.28e-5
CE - BG	.27	-5.30e-5	-1.36e5	2.94e-6

Suppl. Table 6 – Group comparisons on the Stability of IF.
In order, the table reports results from the Levene's test for homogeneity of variance: F-statistics, degrees of freedom (DF) and p value. In the middle, the results from the ANOVA: sum of squares (SS), degrees of freedom (DF), mean squared error (MS), F-statistics and p-value. The error term reports the within-group variation. At the bottom, the results of the pairwise group comparison, with Tukey-Kramer correction. In order, the p-value, observed difference, lower and upper limit.
